# Microstructure and Release Behavior of Alginate–Natural Hydrocolloid Composites: A Comparative Study

**DOI:** 10.3390/polym17040531

**Published:** 2025-02-18

**Authors:** Hatice Sıçramaz, Ali Baran Dönmez, Buse Güven, Derya Ünal, Elif Aşbay

**Affiliations:** Department of Food Engineering, Faculty of Engineering, Sakarya University, 54050 Sakarya, Turkey

**Keywords:** alginate, encapsulation, hydrocolloids, microbeads, release kinetics

## Abstract

This study investigated the effects of combining sodium alginate (ALG) with various natural hydrocolloids on the microstructure and release behaviors of microbeads. The encapsulation solutions were prepared at a 1:1 (*w*/*w*) ratio with ALG as the control and carrageenan (CAR), locust bean gum (LBG), acacia gum (ACA), pectin (PEC), and carboxymethyl cellulose (CMC) as experimental groups. Each formulation contained 0.2% (*w*/*v*) tartrazine and was extruded into a CaCl_2_ solution for bead production. Encapsulation efficiency varied across formulations, with the lowest in the control (ALG-ALG) and highest in ALG-CAR and ALG-CMC, reaching 74% and 78%, respectively. The microbead sizes ranged from 2.07 to 3.48 mm, with the lowest particle diameter observed in ALG-ACA composites. Surface analysis showed smooth and uniform microbeads in the control (ALG-ALG), while ALG-LBG microbeads were rougher. Release kinetics were assessed using various models, with the Higuchi model best describing the release for most formulations (highest R^2^ values). Tartrazine release followed pseudo-Fickian behavior in all formulations, with slower release in ALG-ACA and faster release in ALG-LBG microbeads. This study fills a gap in understanding how the incorporation of different natural hydrocolloids influences both the encapsulation efficiency and release dynamics of alginate-based microbeads, providing valuable insights for applications in food and pharmaceutical industries.

## 1. Introduction

Encapsulation technologies reveal significant importance across various industries, such as food, pharmaceuticals, and biomedical engineering. The key reasons for encapsulation include the need to protect sensitive drugs, bioactive compounds, and other materials from degradation, as well as enhancing their stability and bioavailability. In pharmaceuticals, encapsulation plays a pivotal role in ensuring the controlled and sustained release of drugs [1]. Encapsulation plays a vital role in the food industry by stabilizing probiotics, vitamins, and other bioactive compounds, as well as aroma and color compounds, improving their stability and shelf life [2]. In biomedical applications, encapsulation protects therapeutic agents, such as enzymes and growth factors, and facilitates their targeted, controlled release, thus enhancing their therapeutic efficacy [3]. Polymers, particularly in the form of geopolymers, play a significant role in reducing the environmental pollution caused by effectively encapsulating heavy metals [4].

Hydrogels, which are networks of hydrophilic polymer chains, are widely used in encapsulation due to their ability to encapsulate and release materials in a controlled manner. Among the different types of hydrogels, natural polymers are usually preferred over synthetic alternatives due to their availability, low cost, renewable nature, reduced toxicity, as well as their biocompatibility and biodegradability [5]. One of the most commonly utilized natural polymers in hydrogel formation is alginate, a polysaccharide derived from brown algae. Alginate-based hydrogels are especially favored due to their ability to form strong gels in the presence of divalent cations, such as calcium ions, which makes them highly effective in controlled release systems and encapsulation technologies [6]. Alginate forms strong gel matrices through the well-known “egg-box” model, where calcium ions interact with the guluronic acid blocks in the alginate chain, creating a stable hydrogel structure [7]. The gelation process is achieved by extruding an alginate solution through a nozzle into a cross-linking bath with divalent cations, typically calcium. Calcium ions diffuse into the alginate matrix, forming cross-links between chains, which is essential for stable microbead formation [8]. These microbeads are widely used in controlled release and encapsulation due to their structural stability under varying conditions [9]. The density and distribution of cross-links within the alginate matrix can significantly influence the mechanical properties, swelling behavior, and release kinetics of the encapsulated substances. Higher cross-linking density typically results in a more rigid and less permeable microbead system, which can be advantageous for applications requiring the sustained release of bioactive compounds. Conversely, a lower density of cross-links may enhance the bead’s swelling capacity and permeability, allowing for quicker release profiles, which can be beneficial in certain therapeutic contexts [10]. Research into optimizing the cross-linking parameters is essential to design hydrogel microbeads for specific applications.

Alginate beads not only possess the advantage of being applicable in both in vitro and in vivo environments but also exhibit photophysical properties that respond to imaging systems. These characteristics enable the observation of the transport of loaded biochemical materials within in vivo settings [11]. Recent studies have focused on optimizing the interactions between alginate and various cross-linking agents to improve the mechanical properties and functional performance of alginate-based microbeads. In this context, the alginate-based core-shell structure successfully protected the encapsulated pepsin, retaining 90% of its activity even after 15 days [12]. However, when used alone, alginate hydrogels have certain limitations, such as low mechanical strength in response to environmental changes [13], poor barrier properties of the pores [14], the necessity for enhanced thermal stability [15], and relatively fast release rates [16]. To overcome these limitations, the incorporation of other natural hydrocolloids, such as carrageenan, pectin, locust bean gum, and acacia gum, into alginate-based systems has been extensively studied for multiple purposes [17].

In the literature, plant-origin hydrocolloids have been extensively studied as composite materials due to their natural characteristics. The alginate–carrageenan composite microbeads formulated for the encapsulation of leucine aminopeptidase exhibited a higher encapsulation efficiency and swelling degree compared to alginate alone, resulting in a softer, more elastic, and porous structure [18]. A reduction in anthocyanin degradation [19], increased stability of probiotics [20], and improvement of mechanical properties and thermal stability [21] have been observed in studies using pectin copolymers. The storage stability and release behavior of phenolic compounds were improved by the incorporation of acacia gum into alginate microbeads [22]. The bioactivity of probiotics and antioxidants encapsulated in the carboxymethyl cellulose—alginate copolymer was enhanced by the controlled release during the storage of functional orange juice [23]. The composite microbeads formulated with alginate and locust bean gum exhibited an extended drug release profile [24].

There are many studies in the literature that investigate the properties of alginate-composite hydrogel materials. However, there is no experimental study providing a comprehensive comparison of these natural hydrocolloids with each other. The objective of this study is to characterize the alginate–natural polysaccharide hydrocolloid composite microbeads in terms of their particle size distributions, encapsulation efficiencies, and morphological structures and to determine the release kinetics in this context.

## 2. Materials and Methods

### 2.1. Materials

Sodium alginate, pectin, locust bean gum, carrageenan, and acacia gum were purchased from Smart Chemistry (İzmir, Turkey). Carboxymethyl cellulose, calcium chloride (CaCl_2_), and all other chemicals were employed from Sigma Aldrich (St. Louis, MO, USA). Tartrazine was supplied from Krk Gıda (İstanbul, Turkey).

### 2.2. Encapsulation of Tartrazine

Encapsulation solutions were prepared using 2% (*w*/*v*) hydrogel materials in distilled water. The hydrogel materials were used in combination with sodium alginate (ALG) in a 1:1 (*w*/*w*) ratio. These materials consisted of six distinct groups: ALG as the control, and carrageenan (CAR), locust bean gum (LBG), acacia gum (ACA), pectin (PEC), and carboxymethyl cellulose (CMC) as the experimental groups. In the subsequent sections of the study, the composites have been referred to by the abbreviations of the hydrocolloids used. The food coloring—tartrazine—was added to the hydrogel solutions at a concentration of 0.2% (*w*/*v*). The mixtures were then stirred at 50 °C for 20 min using a magnetic stirrer to ensure uniform dispersion. Subsequently, the hydrogel solutions were extruded from 10 cm height into a 0.1 M CaCl_2_ solution using a 21-gauge (0.514 mm inner diameter) injector and a pump (ABK, Elsan Elektrik, Balıkesir, Turkey) at a flow rate of 5 mL/min under continuous agitation at 800 rpm at 25 °C. Gentle stirring on a magnetic stirrer at 300 rpm at 25 °C was maintained for 60 min for further solidification. The resulting microbeads were subsequently filtered through white filter paper and washed with distilled water to remove excess CaCl_2_ from the outer surface. The experimental procedures were replicated twice in independent batches. The obtained microbeads were refrigerated at 4 ± 2 °C until further analyses.

### 2.3. Physical Properties of Hydrogel Solutions and Microbeads

The viscosities of hydrogel solutions were measured using a Fungilab viscometer (Spain) equipped with an R6 spindle at a speed of 60 rpm. The particle size of microbeads was determined by digital calipers. Moisture contents were analyzed gravimetrically by drying the particles. Texture analyses were conducted using a Brookfield CT3 texture analyzer (Brookfield Engineering Lab, Madison, WI, USA). The texture profile analysis (TPA) method was employed with a target distance of 1.5 mm, using a 25.4 mm diameter cylindrical probe moving at a test speed of 1 mm/s and a trigger force of 0.5 g to obtain hardness (in g) and springiness (in mm) of the microbeads. After entering the dimensions of each sample into the device, a second texture analysis method was applied using the same probe with 50% deformation on the beads while maintaining the same test speed and trigger force. The results were recorded as load (g).

### 2.4. Characterization of Microbeads

The shapes and surface properties of the microbeads were examined using an inverted microscope (Olympus IX81, Tokyo, Japan) and a scanning electron microscope (SEM) (Quanta FEG 250, FEI, Hillsboro, OR, USA) equipped with large field detector (LFD) imaging under low vacuum conditions at 10.0 kV. Molecular interactions between the hydrogel materials were characterized using Fourier transform infrared (FTIR) spectroscopy (PerkinElmer, Spectrum Two, Waltham, MA, USA) in the spectral range of 400–4000 cm^−1^. Differential scanning calorimetry (DSC) was employed for monitoring the thermal stability of the samples. The DSC assays were carried out with a Netzsch DSC 200 F3 Maia (Selb, Germany). Samples ranging from 25 mg to 30 mg were enclosed in hermetically sealed aluminum capsules and subjected to a nitrogen flow of 40 mL/min. The temperature range scanned was from 25 °C to 300 °C at a heating rate of 5 °C/min.

### 2.5. Encapsulation Efficiency

The encapsulation efficiency (EE) of microbeads was assessed indirectly. Particularly, the amount of food coloring entrapped in the microbeads was calculated by the difference between the total coloring incorporated in the encapsulation solution and the quantity of non-entrapped coloring remaining free in the CaCl_2_ solution after microbead formation. The quantity of non-entrapped tartrazine was measured using a calibration curve obtained from absorbance measurements of free tartrazine at 416 nm, taking into account the remaining volume of the CaCl_2_ solution as a dilution factor. The unloaded microbeads (plain beads) were also subjected to the same procedures as a blank analysis. The EE percentage of the microbeads was calculated according to Equation (1) as follows:

(1) $$\mathrm{EE}\%\;=\left[\left(\mathrm{Total}\;\mathrm{colorant}\;-\;\mathrm{Free}\;\mathrm{colorant}\right)\;/\;\mathrm{Total}\;\mathrm{colorant}\right]\times 100$$

### 2.6. Release Kinetics

To assess the release kinetics of food coloring tartrazine from the microbeads, 1 g of beads was immersed in 5 mL of distilled water, and the mixtures were continuously shaken in a water bath at 37 °C during the analysis. At specific intervals, absorbance measurements were taken using UV-Vis spectrophotometry at a wavelength of 416 nm to quantify the released amounts of tartrazine. The absorbance data provided were converted to concentration using a calibration curve obtained from measurements of free tartrazine. The release profile was determined by monitoring the changes in absorbance over cumulative releases (CR) calculated using Equation (2):

(2) $$CR\;\%=\frac{Q_{t}}{Q_{e}}\;.\;100$$

where *Q_t_* represents the cumulative amount of food coloring released at time *t*, and *Q_e_* is the equilibrium amount. The experiments were conducted in triplicate, and the data obtained from this study were presented as the mean value ± standard deviation. The release kinetic data of microbeads have been incorporated into the zero-order (Equation (3)), first-order (Equation (4)), Higuchi (Equation (5)), and Korsmeyer–Peppas (Equation (6)) equations, as follows:

(3) $$Q_{t}=Q_{0}-k_{0}t$$

where *Q*_0_ is the initial amount of tartrazine, *k*_0_ is the zero-order release rate constant expressed in units of concentration/time, and *t* is the time in hours.

(4) $$\ln\frac{Q_{t}}{Q_{0}}=-kt$$

where *k* is the first-order release rate.

(5) $$Q_{t}=k_{H}\;\cdot \;t^{1/2}$$

where *k_H_* is the Higuchi constant reflecting the design variables of the system.

(6) $$\frac{Q_{t}}{Q_{\infty }}=k_{K}t^{n}$$

where $\frac{Q_{t}\;}{Q_{\infty }}$ is the fraction solute release, *k_K_* is a kinetic constant characteristic of the system, and *n* is the kinetic exponent reflecting the release mechanism.

## 3. Results and Discussion

### 3.1. The Physicochemical Properties of Hydrogel Solutions and Microbeads

The physicochemical properties of the microbeads were evaluated in terms of the viscosities of hydrogel solutions, moisture content, particle size, hardness, and springiness values of the microbeads. The viscosity of the ALG-LBG hydrogel solution was 9.90 P, which was significantly higher than the control sample and the other solutions (Table 1). Subsequently, the control sample—ALG-ALG solution—exhibited a viscosity of 1.35 P. The viscosities of the other hydrogel solutions ranged from 0.05 to 0.95 P. The solutions with the lowest viscosities were those produced using ACA and PEC.

The physicochemical properties of microbeads are also given in Table 1. The particle size of the microbeads indicated a positive correlation with moisture content (*r* = 0.64). Specifically, the highest moisture content was observed in ALG-CAR microbeads, which also had the largest diameter. Moisture content was lowest in the ALG-ALG (the control) and ALG-ACA microbeads. The ALG-ACA was also observed to have the lowest particle size among all products. The lower viscosity and lower particle sizes of ALG-ACA and ALG-PEC indicated a reduced water absorption capacity compared to other materials. When examining the texture results of microbeads, significant correlations with moisture content were observed. An increase in water content led to a decrease in hardness (*r* = −0.87) and an increase in springiness (*r* = 0.75). Specifically, ALG-ALG (the control) and ALG-LBG, which had the lowest moisture content, exhibited similar textural values with hardness ranging from 1706 to 1754 g and springiness from 7.1 to 7.2 mm. In contrast, ALG-CAR microbeads, which had the highest moisture content, showed the lowest hardness (311 g) and the highest springiness (11.9 mm). Sensory observations also supported these results (not included in the table as the sensory assessment was based on qualitative evaluations and did not contain numerical data). The load results recorded by 50% deformation of microbeads showed an opposite correlation with hardness (*r* = −0.73). The most elastic sample, ALG-CAR, which displayed the lowest hardness, exhibited the highest load (199.9 g) for 50% deformation. The ALG-CMC was the following, with a load of 116.1 g. The ALG-ACA microbeads, which were both hard and elastic but also the smallest among the samples, were the easiest to deform. When examining the textural similarities between the samples, ALG-ALG and ALG-LBG exhibited similar properties, while ALG-CAR demonstrated the opposite characteristics. ALG-PEC and ALG-CMC also showed relatively similar properties to each other.

Low viscosity in alginate hydrogels has been previously demonstrated to enhance water mobility, promoting Ca^2+^ diffusion and resulting in a higher degree of cross-linking. It has also been reported that increased cross-linking can lead to reduced thickness and improved elasticity [25]. In our study, ALG hydrogels incorporated with ACA and PEC were identified as the two hydrogel composites with the lowest viscosity, and their microbeads obtained through extrusion into CaCl_2_ exhibited the smallest size. In addition, while ALG-CAR demonstrated the highest elasticity, ALG-ACA and ALG-PEC followed closely. These findings, where the lowest viscosity correlated with the highest springiness, confirmed the earlier hypothesis of Niu et al. [25].

### 3.2. Morphology of Microbeads

The diameters of microbeads are influenced by various factors such as molecular weight and charges of hydrogels, nozzle diameter, injection height, stirring speed, duration in CaCl_2_ solution, etc. In clinical applications, it is essential for microbeads to be small in size and uniform for ease of injection into patients and to optimize the controlled release of the active compounds [26]. In food applications, the controlled and targeted release of the active compound from microbeads has a significant impact on its bioavailability and stability [2]. Furthermore, the surface morphology, including the presence and size of pores, is also critical in determining the release kinetics [27]. Therefore, it is important to know the microstructures of the produced microbeads.

**Figure 1:**
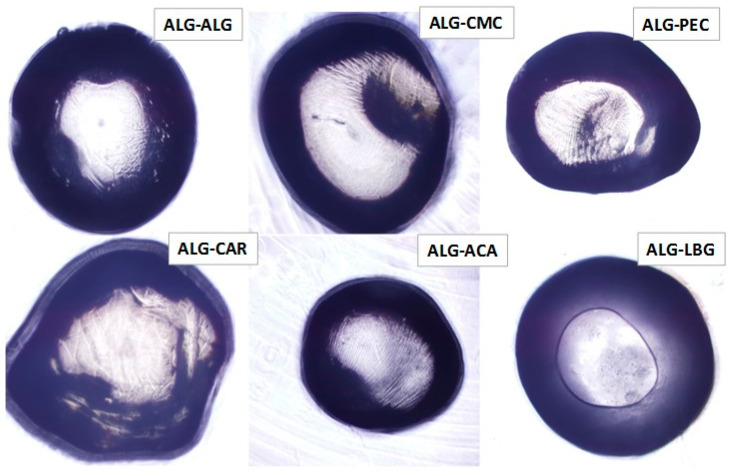
The optical microscopic images of alginate microparticles at a magnification of 4×.

The microstructures of the beads observed under the optical microscope are depicted in Figure 1. The images revealed that the control (ALG-ALG) microbeads exhibited the most spherical and uniform structure among the experimental groups. A central core surrounded by a homogeneous structure was apparent in the ALG-ALG beads. In the ALG-CMC structure, some irregularities and cracks were observed. Among all the beads, ALG-CAR appeared to be the largest in diameter, while ALG-ACA was the smallest. Furthermore, the composite ALG beads containing ACA, followed by PEC, exhibited a denser structure compared to the control and other experimental beads. This outcome was consistent with previous analysis findings and the hypothesis of Niu et al. [25]. The compact configuration in ALG-ACA and ALG-PEC was expected to contribute to slower release rates, which were identified in subsequent analyses. Among the samples, ALG-LGB was the only microbead exhibiting circular structures. The presence of pores and their relationship with the release profile were investigated through further analyses in this study.

**Figure 2:**
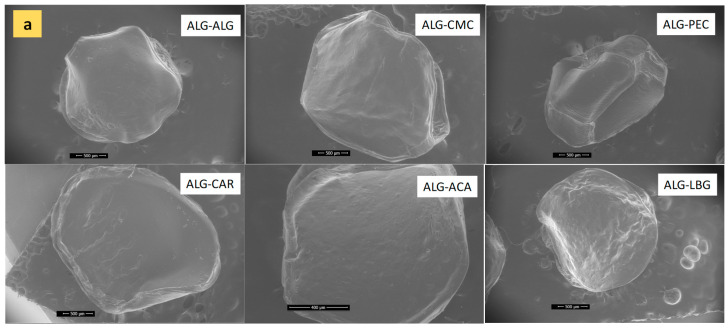

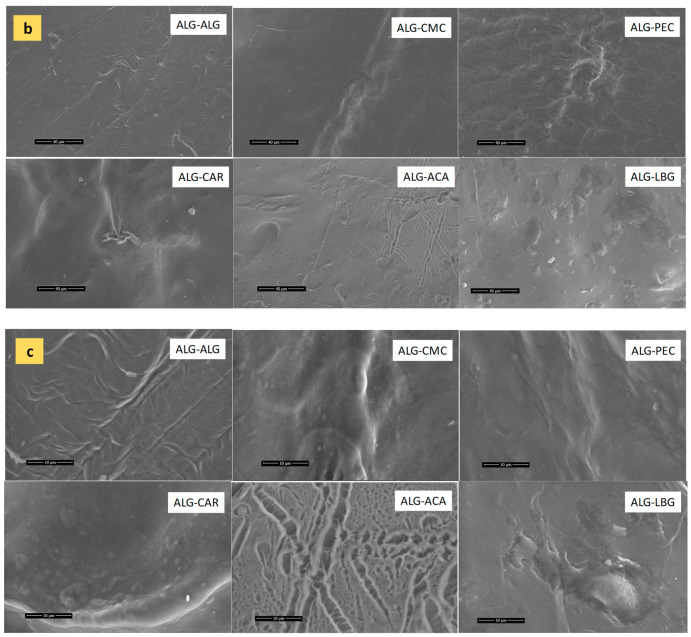
SEM images of microbeads at (**a**) 100× (except ALG-ACA, which was monitored at 250×), (**b**) 2000×, (**c**) 8000×.

The microstructural observations of microbeads under SEM are presented in Figure 2a–c. The particle size measurements observed in the images were consistent with the values given in Table 1 and the optical images in Figure 1. Briefly, the ALG-ACA microbeads presented the smallest diameter compared to the other samples. ALG-ALG microbeads (the control samples) exhibited the most uniform and spherical structure. The ALG-CMC and ALG-PEC microbeads were generally spherical in shape but also exhibited flat regions. Specifically, ALG-PEC displayed diagonal features resembling a rectangular prism. The ALG-CAR, ALG-ACA, and ALG-LBG microbeads revealed oval structures. At high magnifications, a porous structure was not distinctly observed in the products; however, rough textures were present in all products. The roughest surface was observed in ALG-LBG microbeads. This could also indicate an increase in the surface area available for the release of encapsulated material. In a study investigating the most suitable material for the encapsulation of polyphenols, when pectin, cellulose derivatives, and whey proteins were incorporated with alginate, similarly to our results, the smoothest surface was observed in plain alginate microbeads [28].

### 3.3. FTIR

**Figure 3:**
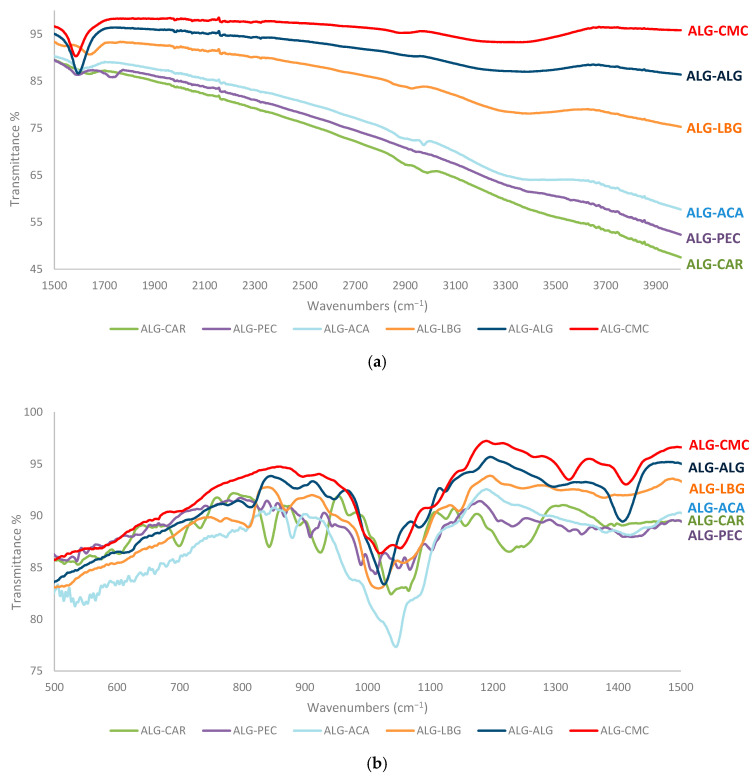
FTIR spectra of microbeads at (**a**) 1500–3900 and (**b**) 500–1500 cm^−1^.

FTIR spectra offer valuable insights into identifying various functional groups present in microbeads and discerning structural differences among hydrocolloids. The FTIR spectra obtained for the tartrazine-loaded hydrocolloid microbeads are presented in Figure 3. A broad O-H stretching band was observed in the range of 3600–3200 cm^−1^. The stretching vibrations of C-H bonds in the range of 3000–2800 cm^−1^ were particularly identified in microbeads excluding pectin. The band observed at 1600 cm^−1^ in ALG-CMC, ALG-ALG, ALG-ACA, and ALG-PEC, and at 1643 cm^−1^ in ALG-CAR and ALG-LBG, corresponds to the asymmetric stretching vibration of the carboxylate groups (O-C-O). ALG-PEC revealed an additional band at 1744 cm^−1^, attributed to the C=O stretching vibration of esterified galacturonic acid [29]. In the range of 1450–1350 cm^−1^, the bands were especially deeper in ALG-ALG and ALG-CMC and smaller in ALG-CAR and ALG-LBG. These bands were in line with the literature and may have originated from the deformations of methylene (CH_2_) or hydroxyl (OH) groups, as well as symmetric stretching vibration of the carboxylate groups (O-C-O) [30,31]. The broadband, particularly intense in ALG-CAR in the range of 1300–1170 cm^−1^, may be attributed to the vibration of O=S=O groups [32] or may originate from the C=O stretching vibrations of carbonyl groups. The band in the range of 1100–1000 cm^−1^ corresponded to the deformation of C-O-C bonds. The presence of two deep bands at 930 and 843 cm^−1^, particularly in carrageenan, indicated the presence of sulfate groups.

**Figure 4:**
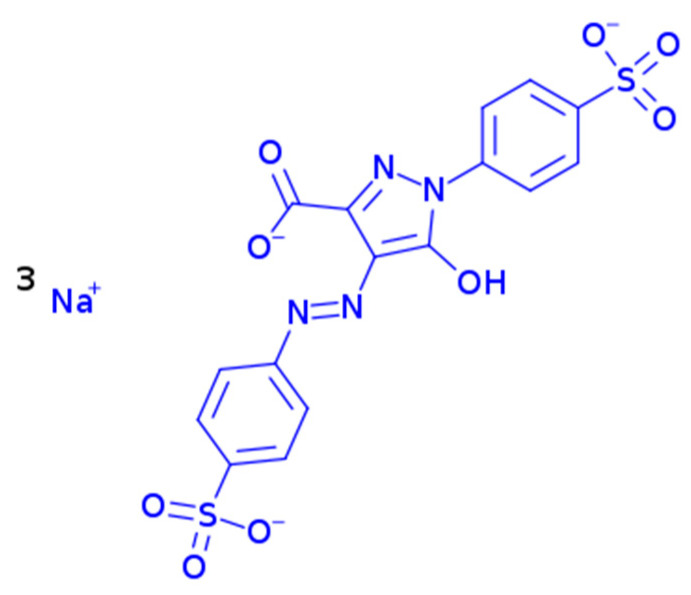
Chemical structure of tartrazine.

Tartrazine is recognized for its sulfonate (SO_3_^−^) groups (Figure 4). The negatively charged sulfonate groups were not expected to form direct ionic bonds with polysaccharide-based hydrocolloids containing negatively charged carboxylate (O-C-O) groups. However, it is possible for the sulfonate groups of tartrazine to interact with the carboxylate groups via Ca^2+^ bridges [33]. Therefore, it was anticipated that the encapsulation efficiencies of ALG-ALG and ALG-CMC, which exhibited distinct carboxylate bands in Figure 3, would be higher due to this indirect ionic binding. Furthermore, in addition to the carboxylate groups, the presence of sulfate groups in ALG-CAR may have enhanced the encapsulation efficiency detected in further analyses through indirect ionic binding.

### 3.4. DSC

**Figure 5:**
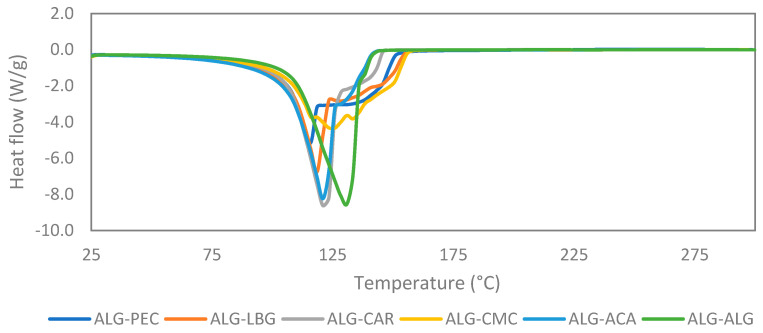
DSC curves of microbeads.

DSC curves provide insights into the thermal properties of materials to determine their potential applications and evaluate the processing parameters. The curves of the microbeads exhibited similar trends with slight variations (Figure 5). All of the samples presented one distinct endothermic peak between approximately 110 °C and 130 °C. The endothermic peaks observed above 100 °C were attributed to dehydration, subsequently followed by decomposition. The precise maximum heat absorption peaks, corresponding to the decomposition temperatures, for ALG-ALG, ALG-CAR, ALG-ACA, ALG-LBG, and ALG-PEC appeared at 130.8 °C, 121.0 °C, 120.9 °C, 118.5 °C, and 116.0 °C, respectively. The ALG-CMC microbeads revealed two overlapping degradation peaks (at 125.9 °C and 133.4 °C), and these peaks were not as sharp as those in other microbeads. This can be attributed to a more heterogeneous structure of ALG-CMC composites. The control microbeads (ALG-ALG) exhibited a single sharp peak due to their homogeneous structure. The incorporation of other hydrocolloids resulted in broader but lower peaks in DSC curves.

The existence of a relationship between DSC curves and the encapsulation properties of microbeads remains unexplained in the literature. Furthermore, Flamminii et al. [34] revealed that while the combination of alginate and pectin exhibited enhanced temperature resistance compared to plain alginate beads, the EE varied depending on the specific properties of the encapsulated material.

### 3.5. Encapsulation Efficiency (EE)

The EE may vary depending on the properties of the encapsulated material. For instance, the solubility of the active compound in the matrix significantly impacts the amount of material that can be effectively encapsulated. Hydrophobic compounds exhibit lower EE in hydrophilic matrices. Additionally, the molecular weight and particle size of the encapsulated material significantly affect its incorporation into the matrix [34]. In the context of composite materials, incorporating various hydrocolloids or polysaccharides can enhance EE by providing synergistic effects [35,36].

**Figure 6:**
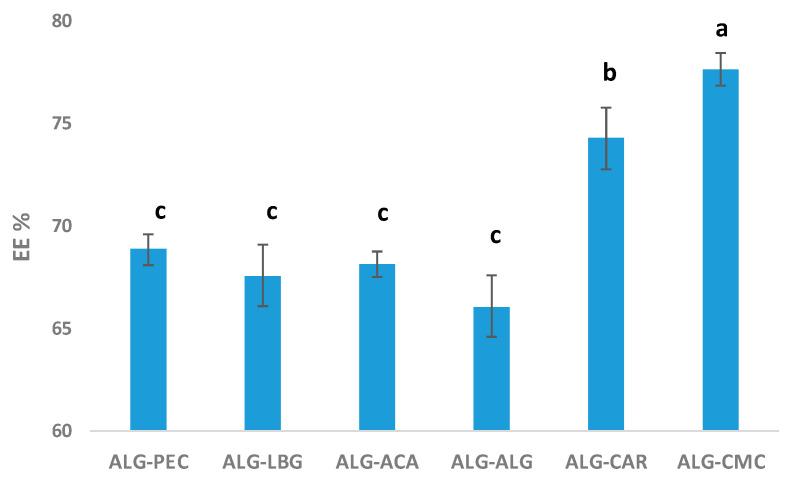
Encapsulation efficiencies (EEs%) of microbeads. Different letters “a–c” represent statistical differences according to Tukey’s test (*p* < 0.05).

Based on the findings of this study, the EE of the microbeads is illustrated in Figure 6. Among the samples, ALG-CMC microbeads exhibited the highest EE at 77.7%, followed by ALG-CAR at 74.3%. The other products revealed similar EEs to the control sample, ALG-ALG. The EEs of ALG-ALG and the other materials ranged 66.1–68.9%. These results indicated that the addition of hydrocolloids, especially ALG-CMC and ALG-CAR, could enhance the EE of ALG-ALG for tartrazine. The higher EE observed for ALG-CMC and ALG-CAR can be attributed to their unique physicochemical properties. In particular, they revealed higher water-binding capacity, which likely forms a more protective gel matrix around tartrazine and enhances EE. The ALG-ALG and ALG-LBG revealed higher viscosities as a solution (before extrusion), but they probably form gels with a more porous structure, leading to slightly higher leakage of water and water-soluble active material—tartrazine. In a study focusing on the encapsulation of food coloring (brilliant blue), researchers achieved a remarkable EE of 92% in ALG-ALG microbeads, in addition to highlighting the significant impact of CaCl_2_ concentration and curing time on EE [37].

### 3.6. Release Mechanism

In many encapsulation systems, delayed and prolonged release profiles are often targeted. In food systems, microbead matrices are investigated to ensure that bioactive compounds resist the gastric environment, reach the intestinal system, and are released there at a specific rate. Additionally, in practical food applications, encapsulation is crucial for a prolonged and delayed release profile for food flavoring and coloring compounds [2].

**Figure 7:**
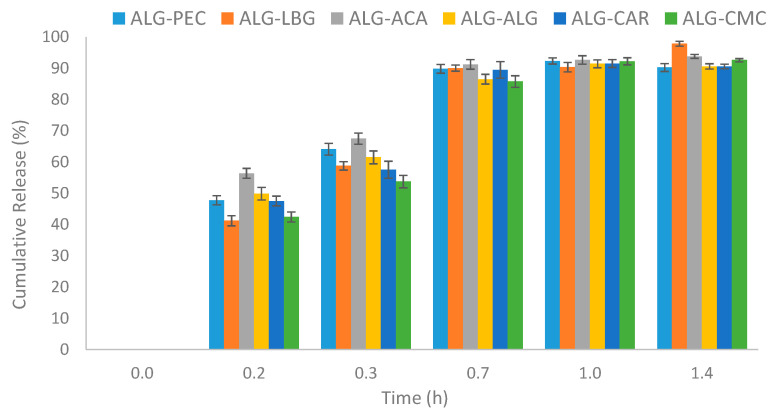
Release profiles of tartrazine from microbeads.

The release profiles of microbeads are presented in Figure 7. All six data series exhibited a sigmoidal curve, where the rate of release changed over time. Initially, there was a rapid release within the first 0.2 h in all samples. This was followed by a more gradual release phase up to approximately 1.0 h and then reaching a plateau with a cumulative release concentration exceeding 90%.

To determine the most suitable kinetic model for tartrazine release from microbeads, the release data were analyzed using zero-order, first-order, Higuchi, and Korsmeyer–Peppas models. The plots of these kinetic models are provided in Supplementary Figures S1–S4. The coefficient of determination (R^2^) values of the linear curve equations are presented in Table 2.

Based on the R^2^ values of the kinetic models (Table 2), none of the samples followed a zero-order release pattern, implying that the release of tartrazine from microbeads was concentration-dependent. The ALG-LBG and ALG-ACA microbeads exhibited release kinetics that were best described by the first-order model, while ALG-ALG, ALG-CMC, and ALG-CAR adhered to the Higuchi model. ALG-PEC’s release profile was consistent with the Korsmeyer–Peppas model. The *n*-kinetic exponent values for all products ranged from 0.12 to 0.20 (see Supplementary Figures S1–S4). In the Korsmeyer–Peppas plot, ALG-ACA exhibited the shallowest slope, whereas ALG-LBG demonstrated the steepest slope. This suggests that the slowest release occurred in ALG-ACA microbeads, while the fastest release occurred in ALG-LBG microbeads among all samples. Furthermore, the diffusional coefficient for tartrazine release indicated a pseudo-Fickian mechanism (n < 0.5) in all microbeads, suggesting both diffusion and matrix relaxation/swelling affect the release.

## 4. Conclusions

This study aimed to improve the encapsulation properties of alginate microbeads by incorporating various natural hydrocolloids. The ALG-ALG (control) and ALG-LBG hydrogel solutions exhibited high viscosity and low water-binding capacity, resulting in rigid, non-flexible microbeads, whereas ALG-CMC and ALG-CAR microbeads displayed greater flexibility. These two composite microbeads also achieved the highest tartrazine encapsulation efficiency, which correlated with their elevated moisture content. In conclusion, the selection of ALG-based composite microbeads should be tailored to meet specific processing requirements. Therefore, plain ALG is ideal for applications requiring high-temperature resistance, while composites incorporated with CMC are recommended for enhanced encapsulation efficiency, LBG for rapid release, and ACA for slow release. This study demonstrated the potential advantages of incorporating hydrocolloids into alginate beads, enhancing encapsulation efficiency and optimizing release profiles, offering significant promise for applications in the food and pharmaceutical industries. Future studies should focus on evaluating the behavior of these composite microbeads within the digestive system.

## Figures and Tables

**Table 1:** Some physicochemical properties of hydrogel solutions and microbeads.

Sample Name	Solution	Microbeads				
	Viscosity (P)	Moisture (%)	Particle Size (mm)	Hardness (g)	Springiness (mm)	Load (g)
ALG-ALG	1.35 ± 0.1 b	97.3 ± 0.07 b	2.66 ± 0.64 abc	1706 ± 71 a	7.2 ± 0.1 c	88.3 ± 6.1 c
ALG-CMC	0.95 ± 0.1 c	97.6 ± 0.33 ab	3.16 ± 0.60 ab	537 ± 6 c	10.4 ± 0.0 b	116.1 ± 9.4 b
ALG-PEC	0.15 ± 0.1 d	97.7 ± 0.21 ab	2.45 ± 0.41 bc	632 ± 22 c	10.3 ± 0.3 b	95.9 ± 12.3 c
ALG-CAR	0.65 ± 0.1 c	98.2 ± 0.30 a	3.48 ± 0.44 a	311 ± 14 d	11.9 ± 0.3 a	199.9 ± 9.6 a
ALG-ACA	0.05 ± 0.1 d	97.4 ± 0.06 b	2.07 ± 0.03 c	1492 ± 54 b	11.0 ± 0.0 b	64.8 ± 11.6 d
ALG-LBG	9.90 ± 0.1 a	97.3 ± 0.01 b	2.78 ± 0.64 abc	1754 ± 29 a	7.1 ± 0.2 c	90.9 ± 7.8 c

ALG-ALG: alginate, ALG-CMC: carboxymethyl cellulose, ALG-PEC: pectin, ALG-CAR: carrageenan, ALG-ACA: acacia gum, or ALG-LBG: locust bean gum hydrogel solutions and microbeads incorporated with ALG. Different letters “a–d” in the same column represent statistical differences according to Tukey’s test (*p* < 0.05).

**Table 2:** The release kinetic model evaluations of tartrazine from microbeads in terms of R^2^.

	ALG-ALG	ALG-CMC	ALG-PEC	ALG-CAR	ALG-ACA	ALG-LBG
Zero-order	0.7145	0.7899	0.6950	0.7272	0.6673	0.7881
First-order	0.8782	0.9282	0.8130	0.8369	0.8750	0.9597
Higuchi	0.8971	0.9378	0.8816	0.9008	0.8650	0.9354
Korsmeyer–Peppas	0.8616	0.8384	0.9058	0.8163	0.8627	0.9081

ALG-ALG: alginate, ALG-CMC: carboxymethyl cellulose, ALG-PEC: pectin, ALG-CAR: carrageenan, ALG-ACA: acacia gum, ALG-LBG: locust bean gum composites of Ca-ALG microbeads.

## Data Availability

The original contributions presented in this study are included in the article/Supplementary Materials. Further inquiries can be directed to the corresponding author.

## Supplementary Materials

The following supporting information can be downloaded at https://www.mdpi.com/article/10.3390/polym17040531/s1, Figure S1: Zero-order kinetics of tartrazine encapsulated in ALG composite microbeads. Figure S2: First-order kinetics of tartrazine encapsulated in ALG composite microbeads. Figure S3: Higuchi kinetics of tartrazine encapsulated in ALG composite microbeads. Figure S4: Korsmeyer–Peppas kinetics of tartrazine encapsulated in ALG composite microbeads.
