# Supplementary material for: Microstructure and Release Behavior of Alginate–Natural Hydrocolloid Composites: A Comparative Study
Source: Polymers (Basel). 2025 Feb 18;17(4):531. doi: 10.3390/polym17040531 (PMC11859929; doi:10.3390/polym17040531)
Supplement: Supplementary file 1 [file polymers-17-00531-s001.zip › polymers-3402804-supplementary.pdf]

Microstructure and Release Behavior of Alginate–Natural Hydrocolloid Composites: A Comparative Study

Hatice Sıçramaz *, Ali Baran Dönmez, Buse Güven, Derya Ünal and Elif Aşbay

Department of Food Engineering, Faculty of Engineering, Sakarya University, 54050 Sakarya, Turkey
* Correspondence: haticesicramaz@sakarya.edu.tr

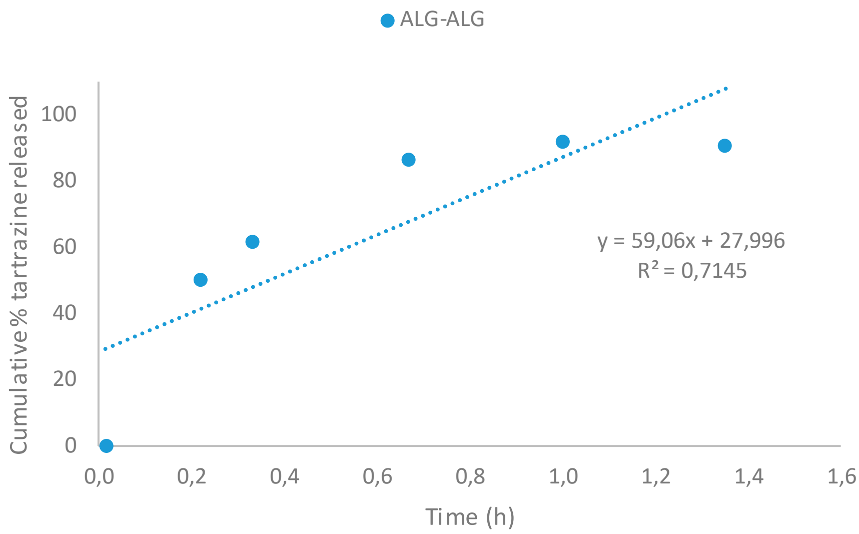

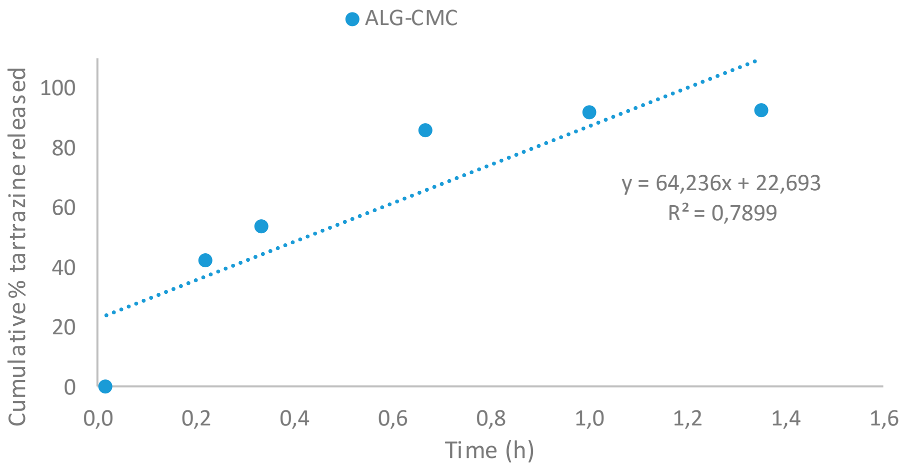

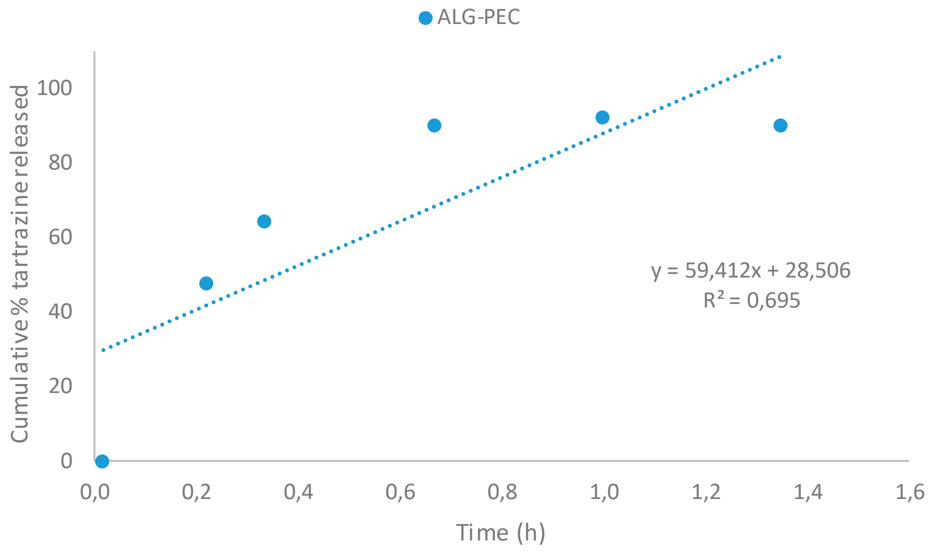
ALG-PEC
Cumulative % tartrazine released
Time (h)
y = 59,412x + 28,506
R² = 0,695

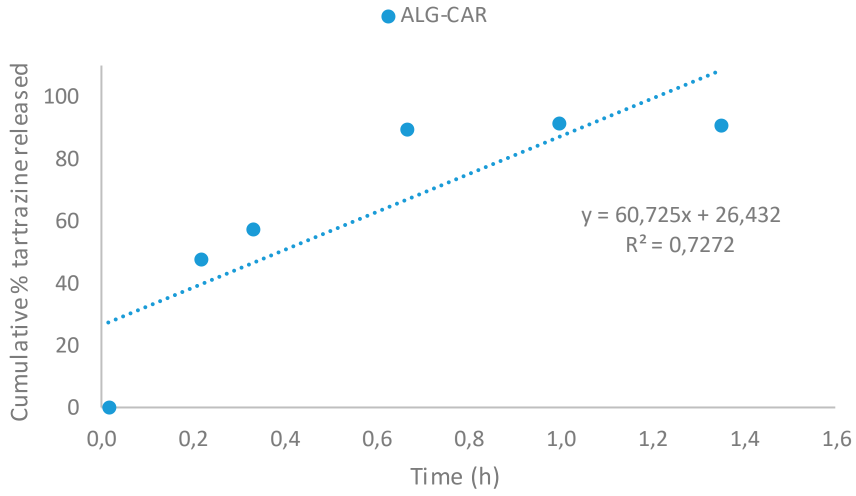
ALG-CAR
Cumulative % tartrazine released
Time (h)
y = 60,725x + 26,432
R² = 0,7272

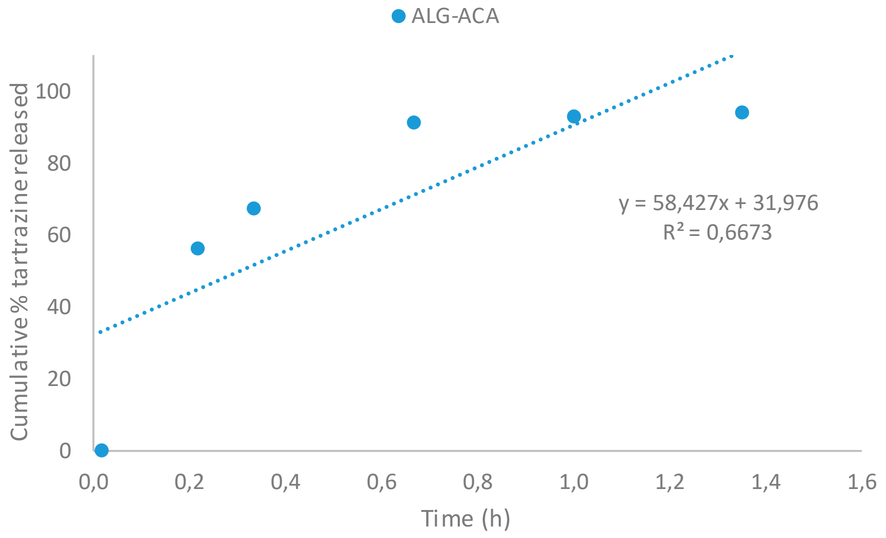
ALG-ACA
Cumulative % tartrazine released
Time (h)
y = 58,427x + 31,976
R² = 0,6673

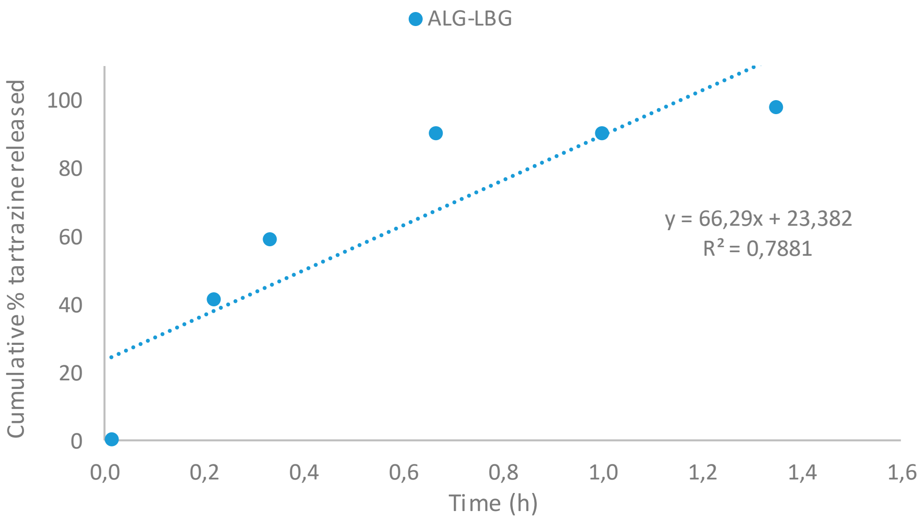

Figure S1. Zero-order kinetics of tartrazine encapsulated in ALG-composite microbeads.

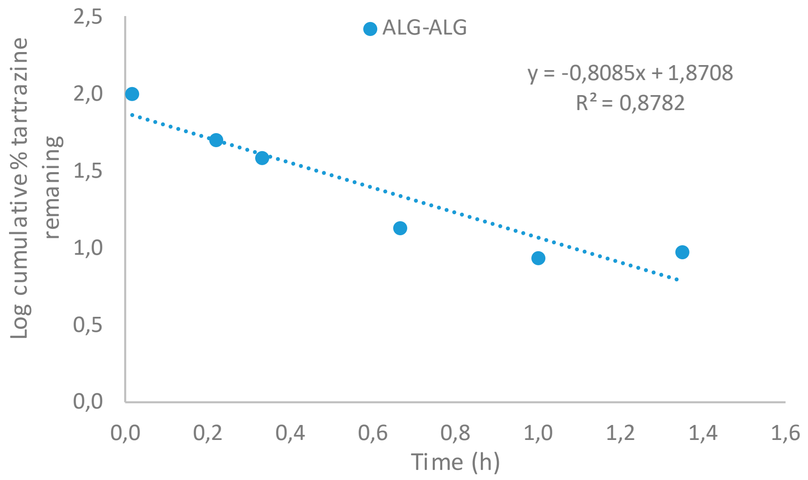

ALG-ALG
y = -0,8085x + 1,8708
R² = 0,8782
Log cumulative % tartrazine remaning
Time (h)
2,5
2,0
1,5
1,0
0,5
0,0
0,0
0,2
0,4
0,6
0,8
1,0
1,2
1,4
1,6

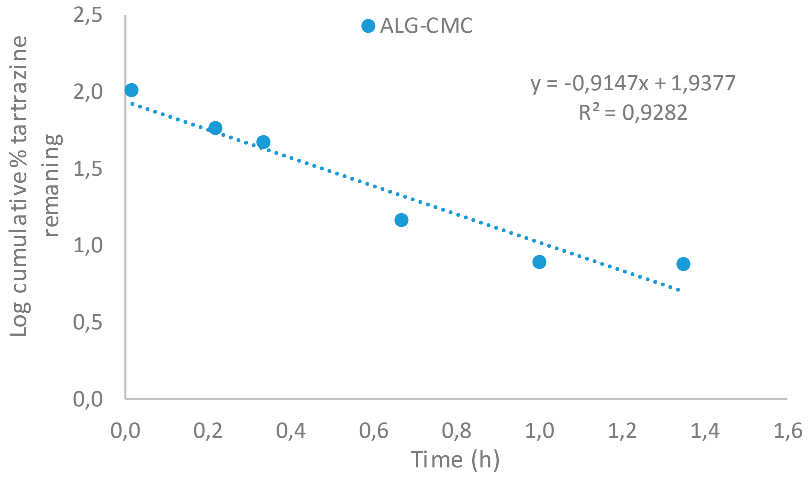

ALG-CMC
y = -0,9147x + 1,9377
R² = 0,9282
Log cumulative % tartrazine remaning
Time (h)
2,5
2,0
1,5
1,0
0,5
0,0
0,0
0,2
0,4
0,6
0,8
1,0
1,2
1,4
1,6

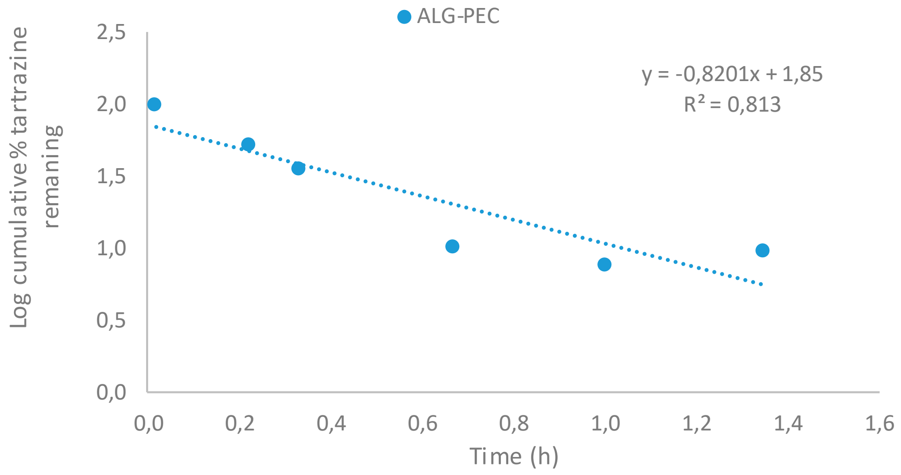

ALG-PEC
y = -0,8201x + 1,85
R² = 0,813
Log cumulative % tartrazine remaning
Time (h)
2,5
2,0
1,5
1,0
0,5
0,0
0,0
0,2
0,4
0,6
0,8
1,0
1,2
1,4
1,6

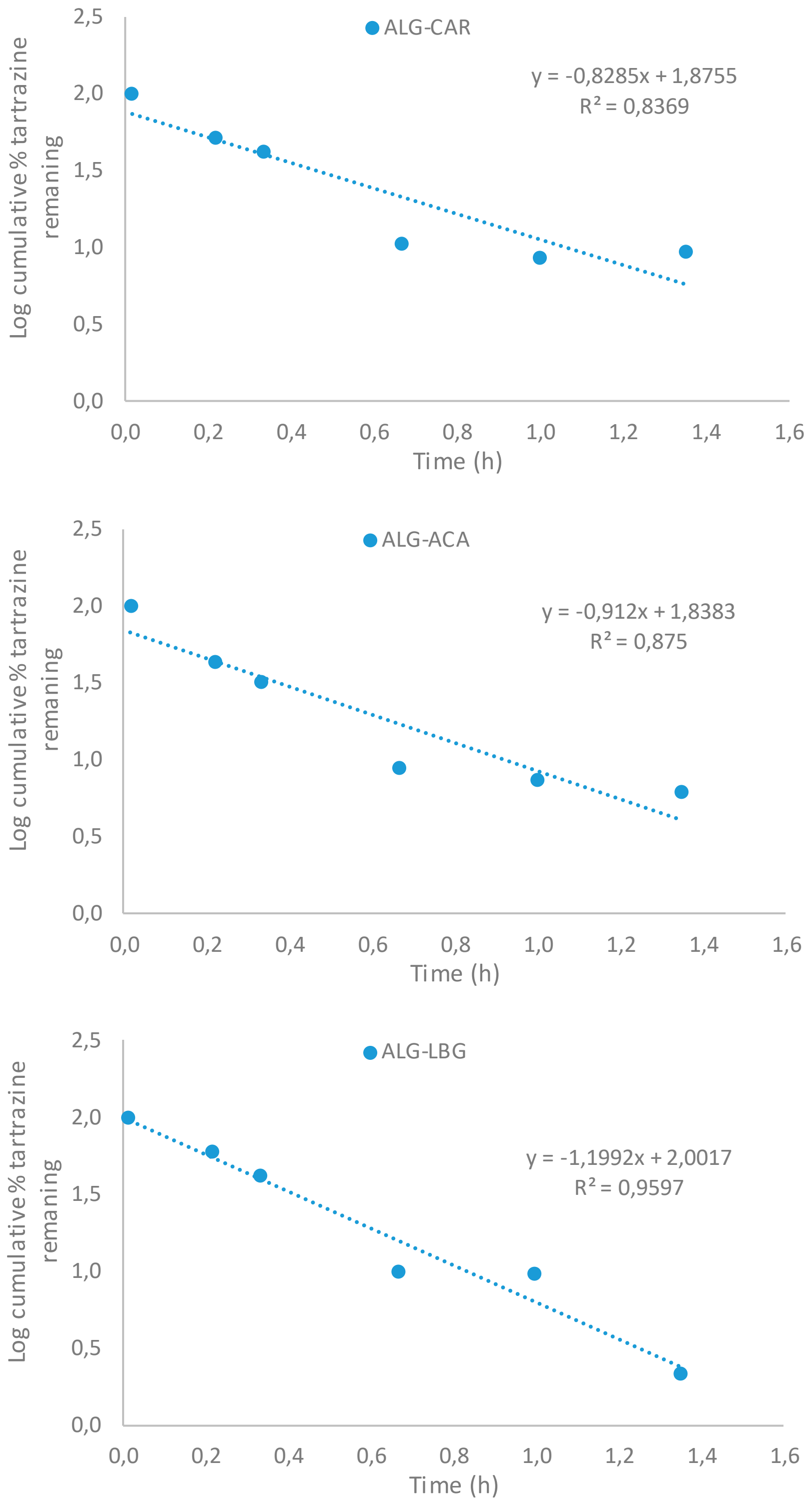

Figure S2. First-order kinetics of tartrazine encapsulated in ALG-composite microbeads.

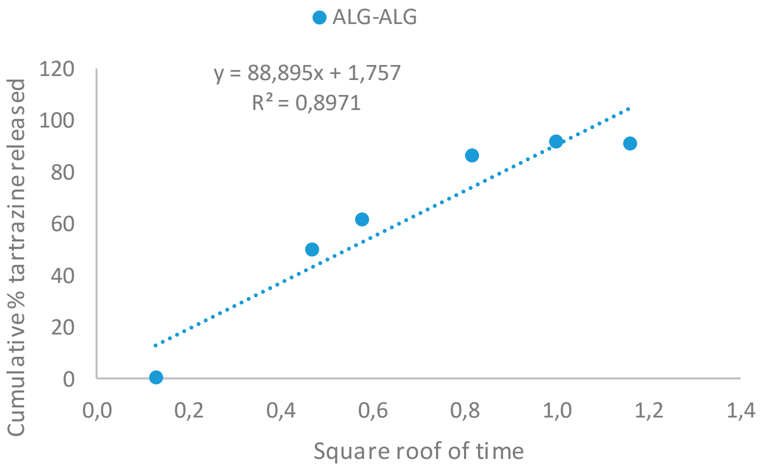
ALG-ALG
y = 88,895x + 1,757
R² = 0,8971
Cumulative % tartrazine released
120
100
80
60
40
20
0
0,0
0,2
0,4
0,6
0,8
1,0
1,2
1,4
Square roof of time

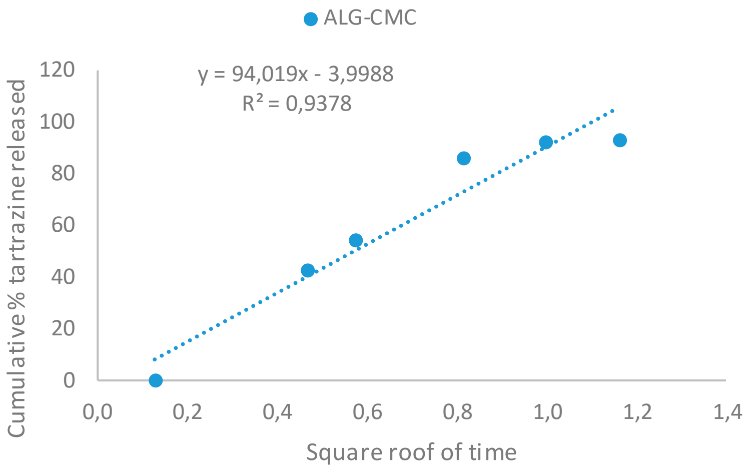
ALG-CMC
y = 94,019x - 3,9988
R² = 0,9378
Cumulative % tartrazine released
120
100
80
60
40
20
0
0,0
0,2
0,4
0,6
0,8
1,0
1,2
1,4
Square roof of time

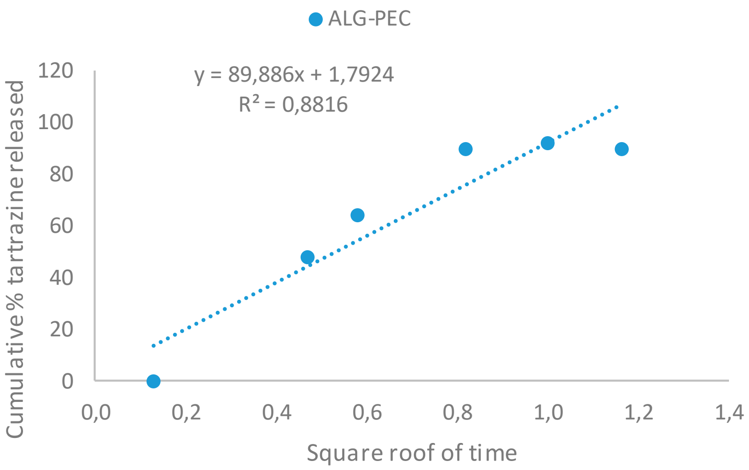
ALG-PEC
y = 89,886x + 1,7924
R² = 0,8816
Cumulative % tartrazine released
120
100
80
60
40
20
0
0,0
0,2
0,4
0,6
0,8
1,0
1,2
1,4
Square roof of time

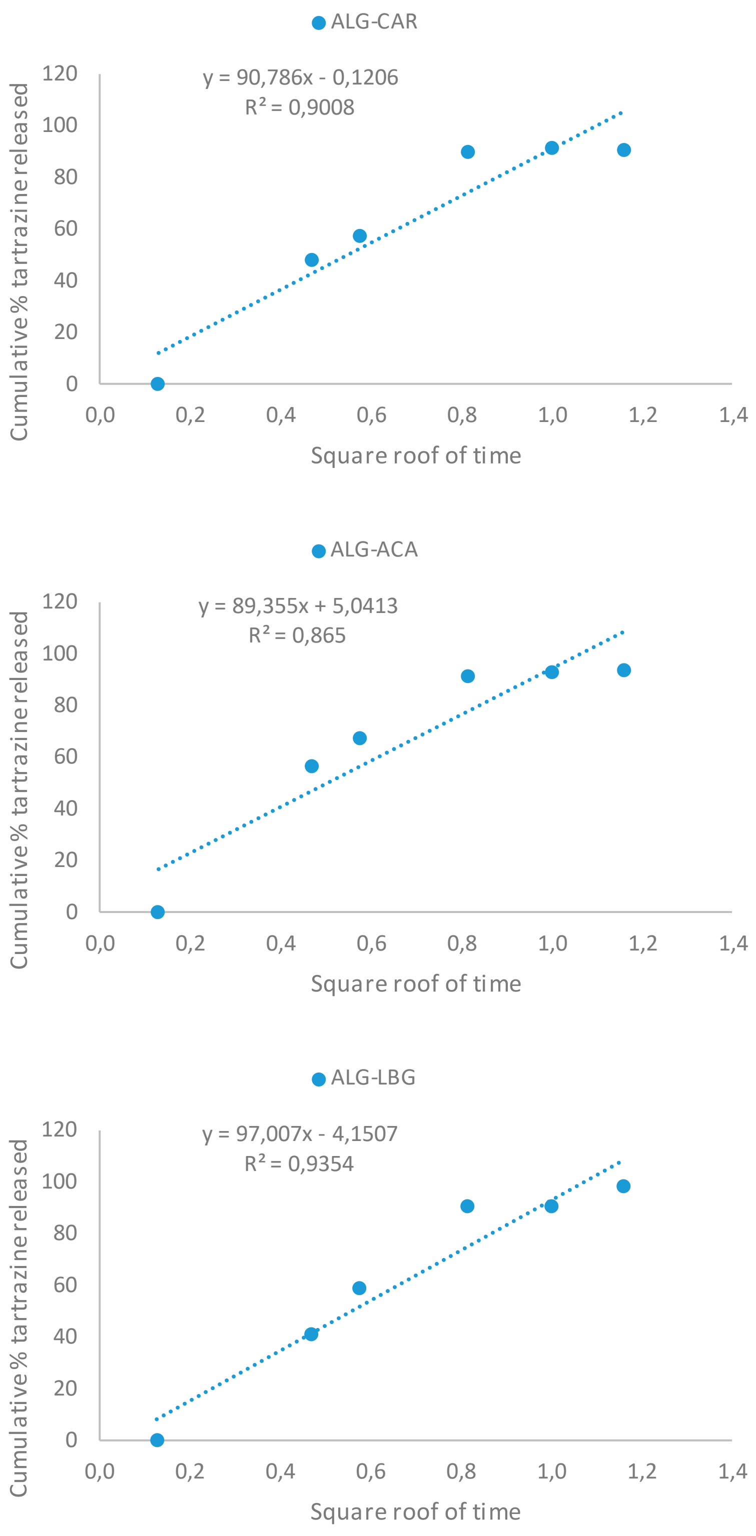

Figure S3. Higuchi kinetics of tartrazine encapsulated in ALG-composite microbeads.

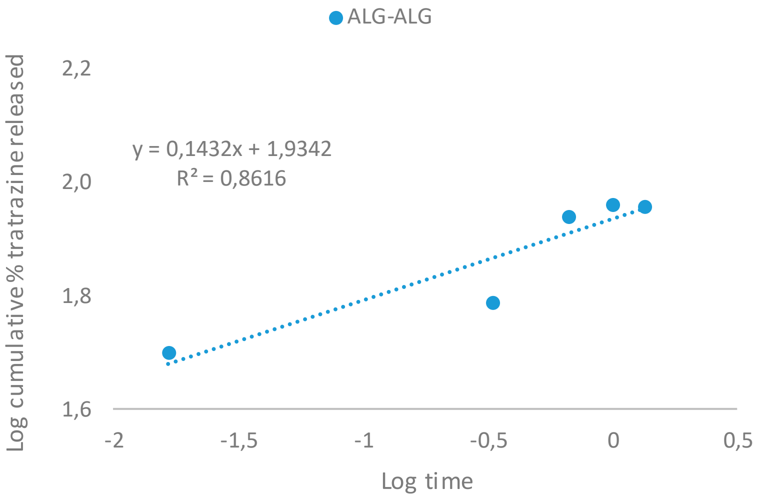
ALG-ALG
y = 0,1432x + 1,9342
R² = 0,8616
2,2
2,0
1,8
1,6
-2
-1,5
-1
-0,5
0
0,5
Log time
Log cumulative % tratrazine released

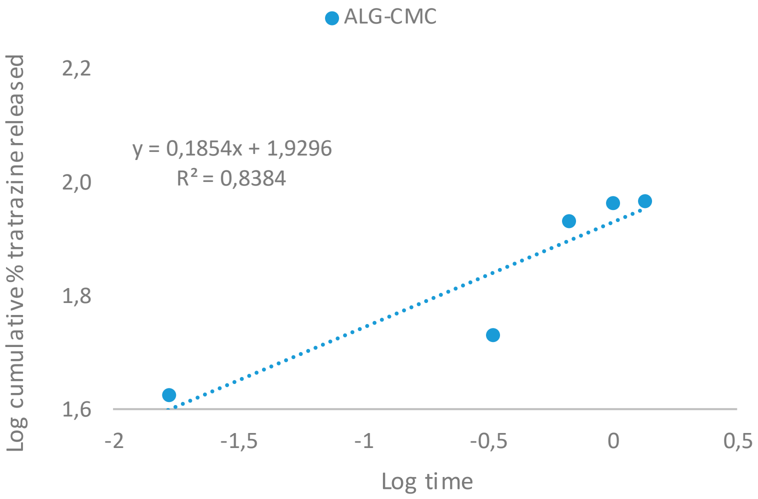
ALG-CMC
y = 0,1854x + 1,9296
R² = 0,8384
2,2
2,0
1,8
1,6
-2
-1,5
-1
-0,5
0
0,5
Log time
Log cumulative % tratrazine released

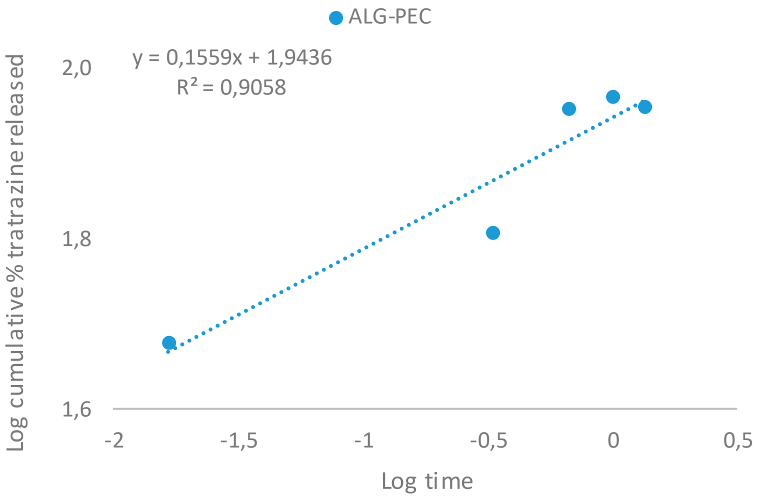
ALG-PEC
y = 0,1559x + 1,9436
R² = 0,9058
2,0
1,8
1,6
-2
-1,5
-1
-0,5
0
0,5
Log time
Log cumulative % tratrazine released

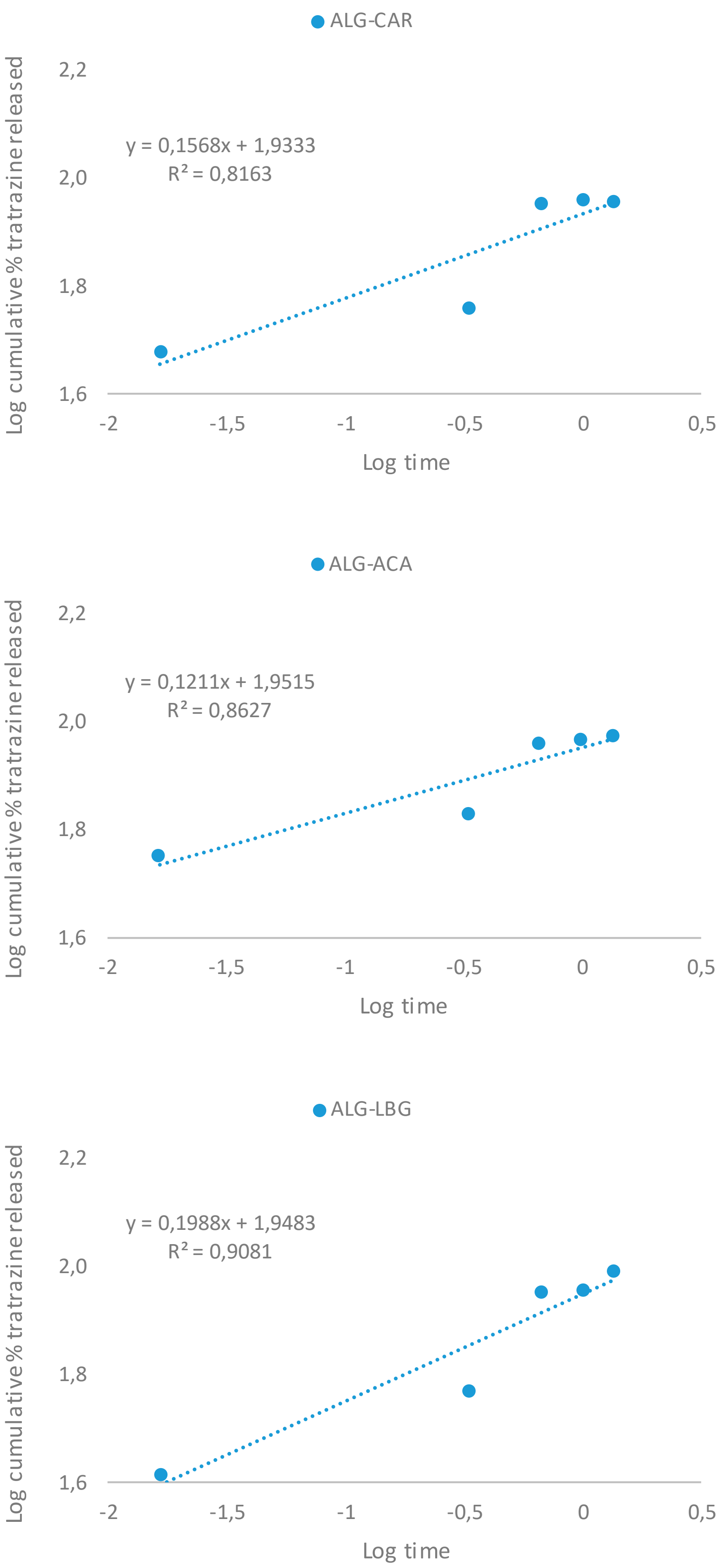

Figure S4. Korsmeyer-Peppas kinetics of tartrazine encapsulated in ALG-composite microbeads.
